# Pulmonary Infection with Influenza A Virus Induces Site-Specific Germinal Center and T Follicular Helper Cell Responses

**DOI:** 10.1371/journal.pone.0040733

**Published:** 2012-07-11

**Authors:** Alexander W. Boyden, Kevin L. Legge, Thomas J. Waldschmidt

**Affiliations:** 1 Interdisciplinary Graduate Program in Immunology, Carver College of Medicine, The University of Iowa, Iowa City, Iowa, United States of America; 2 Department of Pathology, Carver College of Medicine, The University of Iowa, Iowa City, Iowa, United States of America; 3 Department of Microbiology, Carver College of Medicine, The University of Iowa, Iowa City, Iowa, United States of America; Lovelace Respiratory Research Institute, United States of America

## Abstract

Protection from influenza A virus (IAV) challenge requires switched, high affinity Abs derived from long-lived memory B cells and plasma cells. These B cell subsets are generated in germinal centers (GCs), hallmark structures of T helper cell-driven B cell immunity. A full understanding of the GC reaction after respiratory IAV infection is lacking, as is the characterization of T follicular helper (T_FH_) cells that support GCs. Here, GC B cell and T_FH_ cell responses were studied in mice following pulmonary challenge with IAV. Marked GC reactions were induced in draining lymph nodes (dLNs), lung, spleen and nasal-associated lymphoid tissue (NALT), although the magnitude and kinetics of the response was site-specific. Examination of switching within GCs demonstrated IgG2^+^ cells to compose the largest fraction in dLNs, lung and spleen. IgA^+^ GC B cells were infrequent in these sites, but composed a significant subset of the switched GC population in NALT. Further experiments demonstrated splenectomized mice to withstand a lethal recall challenge, suggesting the spleen to be unnecessary for long-term protection in spite of strong GC responses in this organ. Final studies showed that T_FH_ cell numbers were highest in dLNs and spleen, and peaked in all sites prior to the height of the GC reaction. T_FH_ cells purified from dLNs generated IL-21 and IFNγ upon activation, although CD4^+^CXCR5^−^ T effector cells produced higher levels of all cytokines. Collectively, these findings reveal respiratory IAV infection to induce strong T helper cell-driven B cell responses in various organs, with each site displaying unique attributes.

## Introduction

The adaptive immune response to IAV infection is a complex and integrated process, utilizing a range of cell types in defense of the host. After infection, dendritic cells (DCs) migrate from the lung to dLNs where they foster activation and differentiation of CD8^+^ T cells [1]–[5]. IAV-specific CD8^+^ effector T cells migrate to the lung [4], [5] where they receive additional signals from lung-resident DCs [6] in order to eliminate infected epithelium in a Fas receptor, perforin or TRAIL-dependent manner [7], [8]. IAV-specific CD4^+^ T cells also contribute to the primary immune response. In addition to their role in generating T-dependent B cell responses, CD4^+^ T cells become IFNγ-producing T_H_1 cells and cytotoxic effectors that likewise migrate to the lung and aid in resolving the infection [9]–[12]. Abs secreted by B1 B cells participate in protection against and resolution of primary IAV infection as well [13]–[16]. In particular, this B cell subset has been shown to produce natural Abs capable of reacting with IAV [13]–[15] as well as neutralizing IgM Abs generated in a T cell-independent manner after challenge [15]–[16].

While CD8^+^ T cells, CD4^+^ T cells and B1 B cells cooperate to clear a primary IAV infection from the airway, activation of T-dependent B cell responses is central in developing long-term protection from re-infection [17], [18]. In particular, the sustained presence of high affinity switched Abs capable of neutralizing the virus is important in such protection. This was exemplified in the recent 2009 H1N1 epidemic, where older individuals were protected by Abs generated decades earlier through exposure to related H1N1 viruses [19]–[21]. Sustained titers of high affinity Abs result from long-lived antibody forming cells (AFCs) and memory B cells that are induced during a T cell-driven B cell response. Indeed, long-term IAV-specific AFCs and memory B cells have been demonstrated in both the human [22] and mouse [23]–[29]. Importantly, AFCs and memory B cells are products of the GC reaction, a hallmark of T cell-dependent B cell activation. GCs are structures that form within secondary lymphoid organs or ectopic sites after challenge with T cell-dependent antigens, and are driven by specialized CD4^+^ T follicular helper (T_FH_) cells [30]–[32]. Within GCs, B cells undergo intense proliferation and differentiation including class switch recombination, somatic mutation and affinity selection [33]–[35]. These GC processes cooperate to generate a population of selected memory cells and AFCs, which in turn produce high affinity protective Abs for the life of the host [35],[36].

A number of studies have previously examined the T cell-dependent B cell response after primary IAV infection in mice. Many of these reports documented the induction, kinetics and isotype distribution of virus-specific AFCs after respiratory IAV challenge. AFCs were shown to form in the dLNs, lung, NALT and spleen within the first week, and typically peak during the second and third weeks post-infection [25],[26],[37]–[41]. Of interest however, there is isotype- and organ- specific variability in the IAV-reactive AFC response. In all organs, IgM AFCs appear first, peak early and progressively diminish [25],[37]–[40]. At the height of the anti-IAV response, IgG AFCs are dominant in the spleen and dLNs [25],[28],[37]–[40]. Although IgG2 is the predominant subclass among total IgG AFCs, IgG1 and IgG3 also make significant contributions [25],[39]. In the lung, IgG and IgA AFCs are co-dominant at the height of the primary response, with the IgG2 subclass once again constituting the majority of IgG secreting cells [25],[28],[37],[38]. In the NALT, the majority of AFCs secrete IgA at all phases of the response, with contributions from IgG as well [25],[41]. Although memory B cells induced after IAV challenge have not been studied to the same extent, published reports documented their presence in a number of lymphoid organs weeks after infection [28],[29]. Memory B cells were found in the dLNs, lung, NALT and spleen, and similar to AFCs, there was site-specific distribution of IgG and IgA expressing memory cells. Collectively, these studies demonstrate not only T cell-driven activation of IAV-specific B cells in the lung, associated lymphoid tissue and spleen, but specific regulation of this response at each site.

In addition to examining AFCs and memory B cells, a number of reports have also documented the presence of GCs in the dLNs [27],[40],[42]–[44], lung [43],[45]–[47], NALT [43],[48],[49] and spleen [43],[45],[50] after infection with IAV. Although these studies have demonstrated the presence of GCs in various sites upon IAV exposure, none have examined the complete GC response in all organs after infection, nor have they tested for site-specific regulation. Towards this end, the current report examined the GC reaction in dLNs, lung, NALT, and spleen following IAV infection of BALB/c mice. In addition to documenting the kinetics of the response in each site, experiments examined the isotype distribution of GC B cells at all time points. Results demonstrated site-specific control of GC responses with the kinetics, magnitude and isotype distribution of GC B cells differing in each organ. Given the need for T helper cells to drive GC reactions, further studies tested for the presence of T_FH_ cells in various sites after IAV challenge. T_FH_ cells were induced in the dLNs, lung and spleen after infection, and the degree of induction correlated with the size of the GC response. Final experiments examined the cytokines produced by both T_FH_ and CD4^+^ T effector (T_EFF_) cells in dLNs after IAV challenge. Although T_FH_ cells were capable of producing IL-21 and IFNγ, T_EFF_ cells were the major producers of all cytokines including IL-21.

## Materials and Methods

### Ethics Statement

All animal procedures were approved by the University of Iowa Institutional Animal Care and Use Committee (Protocol No. 1005107) in accordance with The Association for Assessment and Accreditation of Laboratory Animal Care, International (AAALAC International) and PHS Animal Welfare (A3021-01) mandates. Accordingly, all possible care was taken to minimize suffering.

### Mice

BALB/c mice were purchased from the National Cancer Institute (Fredrick, MD). Splenectomized and sham-splenectomized BALB/c mice were purchased from The Jackson Laboratory (Bar Harbor, ME). All animals used in experiments were between 8 and 12 weeks of age and housed in the Animal Care Facilities at the University of Iowa.

### Influenza virus infection

For all primary infections, mice were anesthetized with isoflurane inhalation and infected intranasally (i.n.) with a sublethal 0.1LD_50_ (1.07×10^3^ TCIU_50_) dose of mouse-adapted influenza A virus [A/PuertoRico/8/34 (PR8) H1N1] in 50μl of serum-free Iscove's medium. For secondary challenge experiments, sham or splenectomized mice were given a lethal 10.0LD_50_ (1.07×10^5^ TCIU_50_) dose.

### Flow Cytometry

Spleens and dLNs (combined peribronchial and mediastinal) were minced with frosted slides and whole lungs were pressed through a wire mesh to obtain single cell suspensions. The NALT of five individual mice was dissected and pooled as previously described [51]. Cells suspensions were washed with balanced salt solution and subjected to a Fico/Lite-LM (Atlanta Biologicals, Norcross, GA) density centrifugation to obtain viable mononuclear cells, which were then resuspended in staining buffer (balanced salt solution, 5% bovine calf serum, and 0.1% sodium azide). 1×10^6^ cells were subsequently stained utilizing a number of multi-color protocols. Non-specific binding of conjugated Abs was blocked by adding 10μl of rat serum (Pel Freez, Rogers, AR) and 10μg of 2.4G2, an anti-FcγR monoclonal Ab. Rat anti-mouse mAbs used were anti-IgM (b7-6), anti-B220 (6B2), anti-CD44 (9F3), anti-IL2Rα(7D4), anti-CD4 (PerCP/Cy5.5 conjugate, BioLegend, San Diego, CA), anti-CD150 (PE conjugate, BioLegend, San Diego, CA) and anti-CXCR5 (biotin conjugate, BD Pharmingen, San Diego, CA). Goat anti-mouse Abs used were biotin-labeled anti-IgG1, -IgG2a, -IgG2b, -IgG3, and -IgA (all from Southern Biotechnology Associates, Birmingham, AL). FITC-conjugated peanut agglutinin (PNA) was purchased from Vector Laboratories, Burlingame, CA. 2.4G2, b7-6, 6B2, 9F3 and 7D4 were semi-purified from HB101 serum-free supernatants using 50% ammonium sulfate precipitation. b7-6 was conjugated to biotin (Sigma-Aldrich, St. Louis, MO) and 6B2, 7D4, and 9F3 were conjugated to Cy5 (Amersham Pharmacia, Piscataway, NJ) using standard procedures. Purified rat IgG (Jackson Immunoresearch Laboratories, West Grove, PA) was conjugated and used for isotype controls. Primary mAbs or PNA were added to cells and incubated for 20 minutes on ice. For anti-CXCR5 staining, the primary incubation was 30 minutes at room temperature. After washing cells twice in staining buffer, PE-conjugated streptavidin (Southern Biotechnology Associates) was used to detect most biotinylated Abs. PE-Cy7-conjugated streptavidin (eBioscience, San Diego, CA) was used to detect biotin-conjugated anti-CXCR5 mAb. Cells were incubated on ice for 20 minutes and resuspended in fixative (1% formaldehyde in 1.25X PBS) after washing twice with staining buffer. Stained cells were run on a FACSCanto II (Becton Dickinson, San Jose, CA). All data were analyzed using FlowJo software (Tree Star, Ashland, OR).

### IAV-specific ELISA

Whole PR8 virus was added to 96-well ELISA plates (Nalgene Nunc International, Rochester, NY) at 3.2×10^5^ TCIU_50_ per well in 100μl of pH 9.6 carbonate coating buffer and incubated overnight at 4°C. Plates were decanted of virus/coating buffer, blotted, and blocked with 1% BSA solution for one hour at 37°C. Plates were washed three times with PBS-Tween buffer and diluted samples added to the wells in a 100μl volume. Serum samples were added starting at a 1∶200 dilution and bronchioalveolar lavage (BAL) samples starting at a 1∶5 dilution, followed by 1∶2 serial dilutions. Samples were incubated for one hour at 37°C. Plates were washed and various detection antibodies (biotin-labeled goat anti-mouse IgG1, IgG2a, IgG2b, IgG3, and IgA, Southern Biotechnology Associates; biotin-labeled AffiniPure goat anti-mouse IgG, Fc fragment specific, Jackson Immunoresearch Laboratories) were added at a 1∶500 dilution for one hour at 37°C. Plates were washed and a 1∶500 dilution of alkaline phosphatase-streptavidin (Invitrogen, Carlsbad, CA) was added and incubated for one hour at 37°C. Plates were washed a final time, phosphatase substrate (Sigma-Aldrich) added at 2mg/ml and the plates developed for twenty minutes at 37°C. O.D.s were read at 405nm using an EL311 Microplate Autoreader from Bio-Tek Instruments (Winooski, VT). Note that assays measuring different classes of Ab (Figure 4 – total IgM, IgG, IgA or IgG subclasses) were performed in a manner whereby all the serum samples were tested on the same plate at the same time using identical dilutions of the isotype-specific detection antibodies.

### Sorting

dLNs from fifteen mice were harvested and pooled at day 12 post-infection and a viable mononuclear cell suspension prepared as described above. Cells were maintained in azide free balanced salt solution and stained with anti-CD4, anti-CD44, anti-CXCR5 and anti-CD150 as detailed above and sorted on a Becton Dickinson FACSAria II to enrich for three CD4^+^ T cell populations: CD4^+^CD44^lo^ (CD44^lo^) cells, CD4^+^CD44^hi^CXCR5^−^CD150^hi^ (T_EFF_) cells, and CD4^+^CD44^hi^CXCR5^+^CD150^lo^ (T_FH_) cells. Cells were sorted into complete medium consisting of RPMI 1640 (Gibco, Grand Island, NY), 10% endotoxin low fetal calf serum (Hyclone Laboratories, Logan, UT), 5×10^−5^M 2-mercaptoethanol, 1% v/v penicillin-streptomycin and 1% v/v L-glutamine.

### T cell stimulation and cytokine measurements

Sort-purified CD4^+^ T_EFF_, T_FH_, or CD44^lo^ populations were added to 96-well flat bottom plates (Corning Inc., Corning, NY) at 7.5×10^4^ cells per well in 200μl of complete medium. Cells were stimulated at 37°C with PMA (50ng/ml) and ionomycin (1μg/ml) for five or eighteen hours, or with 3μg of plate-bound anti-CD3 (145-2C11) and 15μg of plate-bound anti-CD28 (37.51) mAbs for eighteen hours. Cells were spun down and supernatants were harvested and subjected to cytokine bead array analysis utilizing Milliplex kits (Millipore, Bellerica, MA) as per manufacturer's instructions. Custom bead mixes were used to quantify levels of IFNγ, IL-4, IL-17A and IL-21 protein released by sorted T cells following stimulation. All Milliplex data acquisition and analysis was performed on a Bio-Rad Bio-Plex 200 system (Bio-Rad, Hercules, CA).

### Statistics

Where appropriate, unpaired Student's t test with Welch correction was applied to determine statistical significance between two experimental groups. One-way analysis of variance (ANOVA) tests with Bonferroni post-test was applied when determining the statistical significance between several experimental groups. All statistical analyses were performed with the GraphPad InStat software program (La Jolla, CA).

## Results

### Respiratory IAV infection induces a GC response in multiple sites

**Figure 1:**
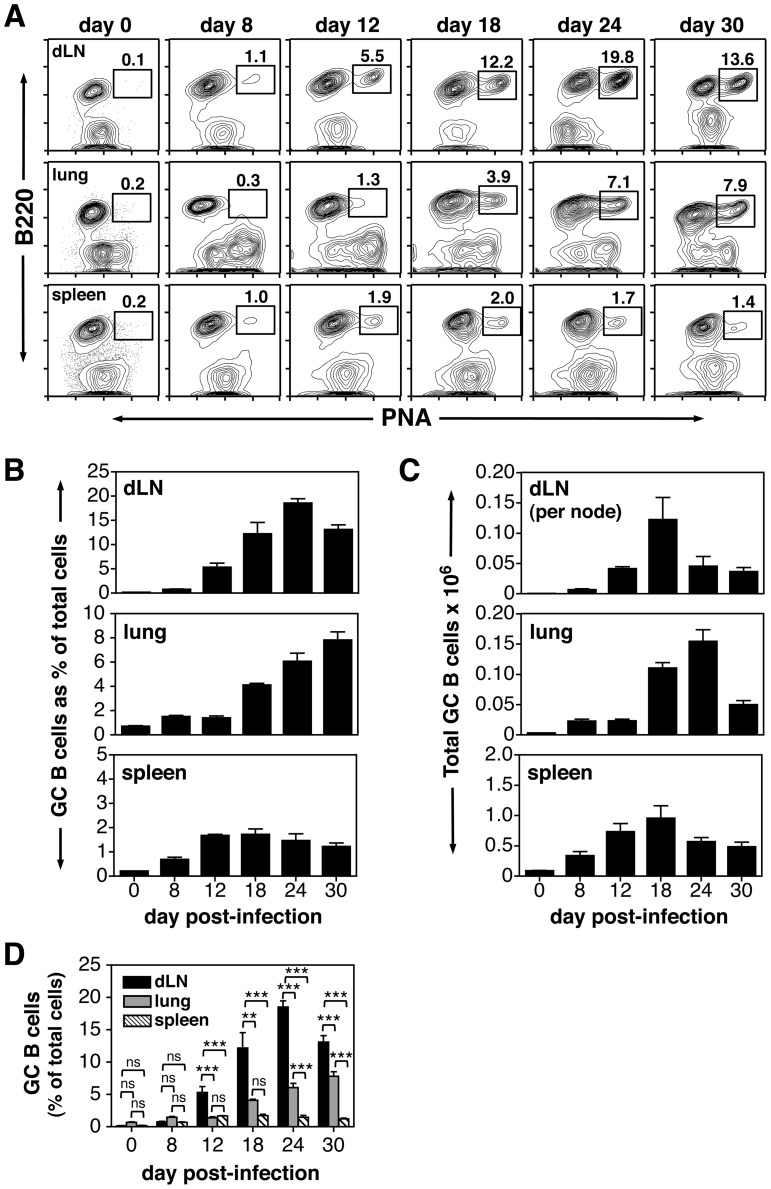
IAV infection induces GC reactions in dLNs, lung and spleen. Animals were infected i.n. with a 0.1LD_50_ dose of IAV on day 0. dLNs, lung, and spleen were harvested on days 8–30 post-infection and stained with PNA and anti-B220 mAb. Tissues from naïve uninfected mice were also analyzed and represent day 0. A) GC B cell populations (gated B220^+^PNA^hi^ cells) from dLNs, lung and spleen are shown at all time points. The value above each gate represents the individual percentage of GC B cells from that sample. B) Bar graphs represent the percent of B220^+^PNA^hi^ GC B cells within the viable lymphocyte-gated population. C) Bar graphs represent the number of total recovered GC B cells per organ. dLN data are shown as total cells per lymph node. D) Bar graphs represent the percent of B220^+^PNA^hi^ GC B cells within the viable lymphocyte-gated population. Each bar represents mean ± SEM. *n* = 5–6 mice per group and time point. ns  =  not significant; **p<0.01; ***p<0.001; determined with the ANOVA test.

Although a number of previous reports demonstrated GC reactions in lymphoid sites and the lung after IAV infection [27],[40],[42]–[50], no one has undertaken an integrated study examining the kinetics and characteristics of the GC response in the dLNs, lung, spleen and NALT. BALB/c mice were therefore given a sublethal dose of IAV (PR8) i.n., and organs were harvested at various time points (days 8–30) post-infection. Single cell suspensions were prepared and analyzed by flow cytometry. In naïve mice, GC (B220^+^PNA^hi^) B cells were not observed in the dLNs (peribronchial and mediastinal), lung and spleen (Figure 1A), and the large majority of resident B cells in these sites exhibited a follicular phenotype (Figure S1). The lack of pre-existing GC B cells in sterile sites (lung) or secondary lymphoid organs that drain sterile sites is expected in naïve specific pathogen-free mice. Upon challenge, robust GC B cell responses were induced in the dLNs, lung and spleen (Figure 1A). GC reactions were not induced in non-draining distal lymph nodes (popliteal nodes) after i.n. infection (data not shown) indicating that spread of viral antigen is limited to the upper respiratory track and spleen. As a percent of total lymphoid cells, the GC response was largest in the dLNs with nearly 20% of recovered cells displaying a B220^+^PNA^hi^ phenotype at the peak of the reaction (Figure 1B and Figure 1D). Although the percentage of GC B cells was lowest in the spleen (Figure 1B and Figure 1D), this organ contained the largest total number of GC B cells due to overall cell recovery (Figure 1C). When examining the kinetics of the GC reaction, as measured by total cell number (Figure 1C), GC B cells appeared by day 8 in all sites, but peaked at different time points post-infection. Whereas the response was at its height at day 18 in both the dLNs and spleen, lung GC B cells were at their peak at day 24 (Figure 1C). Of note, the kinetics of the response differed when comparing GC B cell percentage (Figure 1B) with total GC B cell recovery (Figure 1C). These differences were largely accounted for by attrition in the total non-GC B cell population. Between days 18 and 30, there was a 2.4-fold loss of total B cells in the dLNs, a 4.3-fold loss in the lung and a 1.3-fold loss in the spleen (data not shown).

### GC responses exhibit site-specific isotype switching patterns

**Figure 2:**
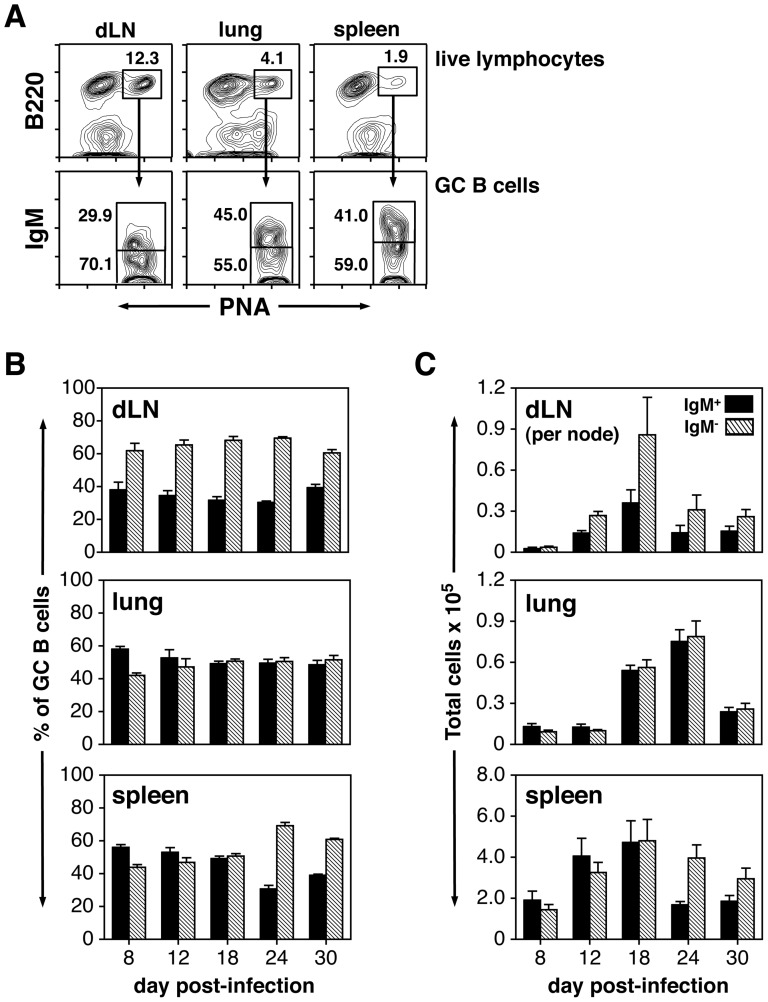
IAV-induced GC B cell responses exhibit site-specific switching characteristics. Animals were infected i.n. with a 0.1LD_50_ dose of IAV on day 0. dLNs, lung, and spleen were harvested on days 8–30 post-infection and stained with PNA, anti-B220 mAb and anti-IgM mAb. A) Representative plots show the gating strategy used to define non-switched IgM^+^ and switched IgM^−^ GC B cells in dLNs, lung and spleen at day 18 post-infection. B) Bar graphs represent the percent of non-switched IgM^+^ (closed bars) and switched IgM^−^ (hatched bars) B cells within the B220^+^PNA^hi^ GC population. C) Bar graphs represent the total number of non-switched IgM^+^ (closed bars) and switched IgM^−^ (hatched bars) GC B cells per organ. dLN data are shown as total cells per lymph node. Each bar represents mean ± SEM. *n* = 5–6 mice per group and time point.

Previous studies from our laboratory demonstrated isotype switching within splenic GCs to be highly regulated [33],[52]–[54]. In particular, splenic GC responses induced to experimental antigens exhibited a steady ratio of IgM^+^ to IgM^−^ switched B cells, with the former constituting at least half of the GC population [33],[52]–[54]. The proportion and number of IgM^+^ and switched B220^+^PNA^hi^ GC B cells were therefore assessed in the dLNs, lung and spleen after respiratory IAV challenge. Figure 2A shows the gating strategy for delineating IgM^+^ and IgM^−^ GC B cells. The percent of IgM^+^ and switched B cells within the GC population, as well as total numbers of each subset are shown in Figure 2B and Figure 2C, respectively. Similar to results obtained from examining the magnitude and kinetics of the IAV-induced GC reaction (Figure 1), the pattern of isotype switching within GCs was site-specific. In dLNs, IgM^−^ switched cells constituted approximately 60% of GC B cells with the remaining 40% displaying IgM. This ratio was steady throughout the entire response (Figure 2B). In the lungs, the pattern was also steady, although IgM^+^ and switched GC B cells were present in equal (50∶50) proportions (Figure 2B). In the spleen however, a dynamic pattern of switching was observed. Whereas IgM^+^ B cells were slightly more numerous early after IAV infection, switched B cells constituted the majority of the GC population late in the response (Figure 2B). Statistical analysis revealed that the proportion of switched (IgM^−^) GC B cells in the dLNs was significantly greater than those within the lung at all time points post-infection (Figure S2). The percentage of switched GC B cells in the dLNs was also significantly greater than those within the spleen from day 8 to day 18 (Figure S2). Additionally, switched GC B cell proportions in the spleen were significantly greater compared to the lung at days 24 and 30 (Figure S2). Overall, the percentage of switched GC B cells in the dLNs was greater than that of the lung and spleen at nearly every time point post-infection (Figure 2B and Figure S2).

**Figure 3:**
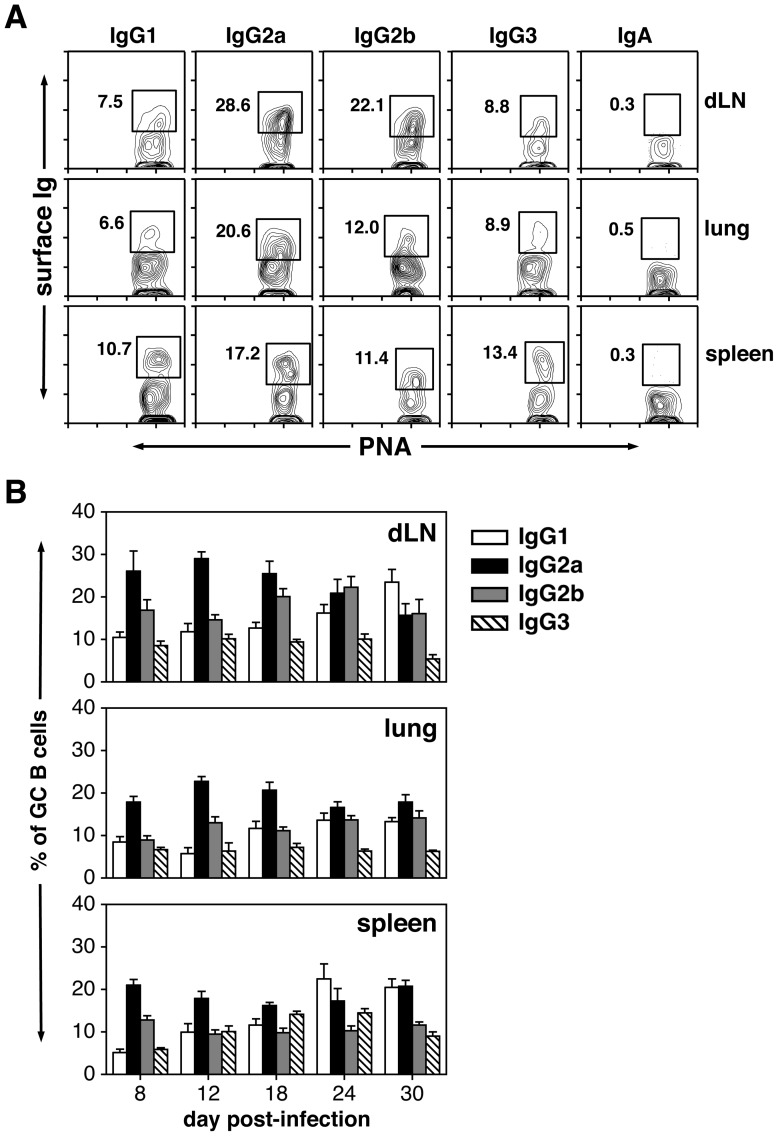
Distribution of IgG subclass-expressing B cells within the GC. Animals were infected i.n. with a 0.1LD_50_ dose of IAV on day 0. dLNs, lung, and spleen were harvested on days 8–30 post-infection and stained with PNA, anti-B220 mAb and either goat anti-IgG1, IgG2a, IgG2b, IgG3 or IgA specific Abs. A) Representative plots (from B220^+^PNA^hi^ parent gates) show the gating strategy used to define IgG1^+^, IgG2a^+^, IgG2b^+^, IgG3^+^ and IgA^+^ GC B cells in dLNs, lung and spleen at day 18 post-infection. B) Bar graphs represent the percent of IgG^+^ subsets within the B220^+^PNA^hi^ GC population. Each bar represents mean ± SEM. *n* = 5–6 mice per group and time point.

Given the different proportions of IgM^−^ switched B cells within the GCs of dLNs, lung and spleen after challenge, the isotype distribution of the switched population in each site was determined. Specifically, the percentage of IgG1^+^, IgG2a^+^, IgG2b^+^, IgG3^+^ and IgA^+^ cells within the B220^+^PNA^hi^ GC population was measured. The gating strategy for these experiments is shown in Figure 3A. Whereas IgG^+^ GC B cells were easily detected in all three organs, IgA^+^ GC B cells were rare at all time points tested (Figure 3A and data not shown). When examining the distribution of switched IgG isotypes within the GC B cell population, it is clear that IgG2^+^ cells (IgG2a + IgG2b) composed the largest percentage of the switched response at most time points in the dLNs, lung and spleen (Figure 3B and Figure S3). Total cell recoveries likewise revealed that the switched GC B cell compartment was dominated by IgG2^+^ cells in the various organs at most time points (Figure S3). Although the percentage of IgG1^+^ GC B cells exhibited a significant increase in the dLNs and spleen as the response matured, this was not found when total cell recoveries were examined (Figure S3). IgG3^+^ GC B cells were clearly present in all sites after IAV challenge, but were a minor constituent whether measuring percentage or total cell recovery (Figure 3B and Figure S3).

### Isotype switched GC B cell populations reflect IAV-specific Ab responses

**Figure 4:**
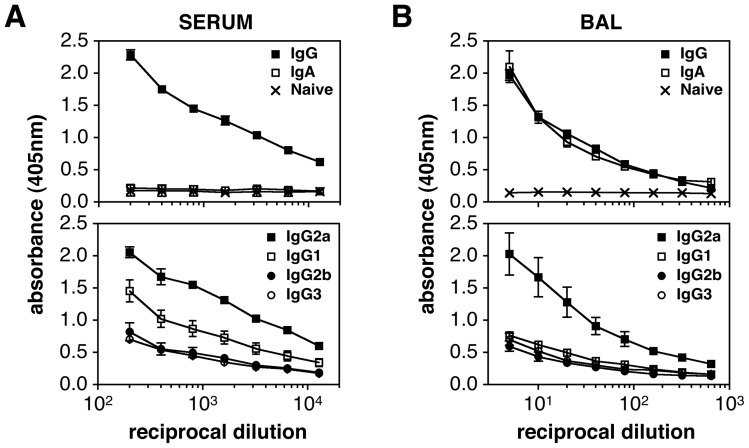
IAV-specific Ab responses in serum and BAL fluid. Animals were infected i.n. with a 0.1LD_50_ dose of IAV on day 0. Serum and BAL samples were harvested from mice on day 24 post-infection or from naïve uninfected animals. Serum and BAL Ab responses were measured using an IAV-specific whole virus ELISA. A) The top panel shows total IAV-specific IgG and IgA responses in serum. IAV-specific IgA levels were near background levels. IAV-specific IgG and IgA Abs were undetectable in sera from uninfected animals. The bottom panel represents IAV-specific IgG1, IgG2a, IgG2b, and IgG3 Ab levels is serum. B) The top panel shows total IAV-specific IgG and IgA responses in BAL samples. IAV-specific IgG and IgA Abs were undetectable in BAL fluid from uninfected animals. The bottom panel represents IAV-specific IgG1, IgG2a, IgG2b, and IgG3 Ab levels in BAL fluid. Each value represents mean ± SEM. The results in panels A and B are representative of 2 separate ELISA tests performed with separate groups of mice. *n* = 3 mice per group.

Examination of GCs in the dLNs, lung and spleen revealed that IgG2^+^ cells represented the largest subset of IgM^−^ switched GC B cells at most time points (Figure 3B and Figure S3). IgG1^+^ and IgG3^+^ B cells composed the remaining switched GC populations, with the percentage of each being dependent upon organ and time point (Figure 3B and Figure S3). Of interest, IgA^+^ GC B cells were infrequent in the dLNs, lung and spleen (Figure 3A). The question arose as to whether levels of IAV-specific switched Abs in the serum and BAL fluid reflected the composition of GC B cell subsets. Serum and BAL were therefore collected from mice at day 24 post-infection and IAV- and isotype-specific ELISAs were performed. As expected, total IAV-specific IgG was plentiful in the serum at day 24, with IgA titers being nearly undetectable (Figure 4A). Examination of IgG subclasses showed IgG2a to score the highest signal in the IAV-specific ELISA, although IgG1, IgG2b, and IgG3 IAV-specific Abs were also demonstrable (Figure 4A). These findings are consistent with previous reports examining IAV-specific serum Abs in BALB/c mice after infection [38],[55]–[57]. Since serum Abs are likely secreted by AFCs generated as a result of GC reactions in the dLNs and spleen, these results are in agreement with the heightened presence of IgG2a^+^ GC B cells throughout most of the response. Determination of IAV-specific Ab levels in BAL revealed high titers of both IgG and IgA (Figure 4B). When testing the subclass distribution of IAV-specific IgG Abs, IgG2a composed the majority of the IgG Ab pool, with IgG1, IgG2b and IgG3 present in lower levels (Figure 4B). These data are also consistent with previous findings [26],[57], but pose a question as to the source of AFCs producing IgA in the lung. Testing for IgA^+^ GC B cells in the lung demonstrated this subset to be rare, suggesting lung IgA AFCs to be generated in another site.

### IgA^+^ GC B cells are present in NALT

**Figure 5:**
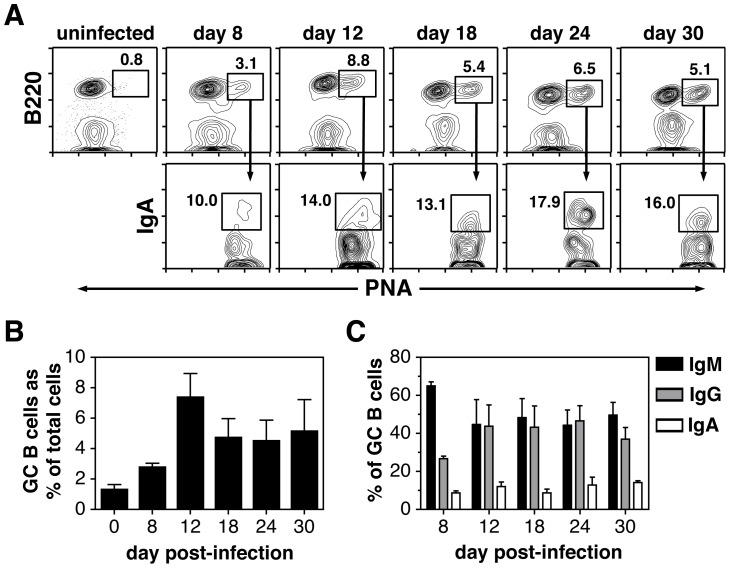
IgA expressing GC B cells are present in NALT. Animals were infected i.n. with a 0.1LD_50_ dose of IAV on day 0. NALT was dissected on days 8–30 post-infection and stained with PNA, anti-B220 mAb and either anti-IgM mAb, a cocktail of goat anti-mouse IgG1, IgG2a, IgG2b, IgG3 specific Abs, or goat anti-mouse IgA specific Ab. Tissues from naïve uninfected mice were also analyzed (day 0). A) Representative plots show the gating strategy used to define IgA^+^ B cells within the B220^+^PNA^hi^ GC compartment at all time points. The value next to each gate represents the individual percentage of GC B cells or IgA^+^ GC B cells from that sample. B) Bar graphs represent the percent of B220^+^PNA^hi^ GC B cells within the viable lymphocyte-gated population. C) Bar graphs represent the number of IgM^+^, total IgG^+^ and IgA^+^ GC B cells. Each bar represents mean ± SEM. *n* = 3 experiments, with each experiment (and time point) containing pooled NALT tissue from 5 mice.

IAV-specific IgA Ab is produced in high titer in the respiratory tract after infection (Figure 4B, [26],[57]) and is important for upper airway protection [58],[59]. Given the paucity of IgA^+^ GC B cells in the lung (Figure 3A), NALT was examined based on previous reports showing this tissue to be enriched for IgA-secreting AFCs after IAV challenge [25],[41]. NALT was therefore harvested from the dorsal side of the pallet [51] at various time points after IAV challenge and subjected to flow cytometric analysis (Figure 5). In naïve mice, B220^+^PNA^hi^ GC B cells were largely absent in NALT and the majority of recovered cells exhibited a follicular B cell phenotype (Figure 5A and Figure S1). Upon IAV infection, GC populations were found in the NALT at all time points tested (Figure 5A). The percent of GC B cells in this site peaked at day 12 post-challenge and remained relatively steady into the second and third week after infection (Figure 5B). (Since NALT is not a discrete anatomic site, dissection of these structures is inherently variable as are total cell recoveries. Total GC B cell numbers were therefore not calculated). When examining the isotype distribution of the GC B cell population, IgA^+^ cells were now easily detected within the B220^+^PNA^hi^ pool (Figure 5A), constituting approximately 10-15% of the GC response at all time points tested (Figure 5C). IgM^+^ B cells dominated the GC population early in the NALT response, with IgM^+^ and IgG^+^ GC B cells present in equal proportions as the GC reaction matured (Figure 5C). These results suggest that NALT may be a significant source of IgA^+^ AFCs in the upper respiratory tract leading to IAV-specific IgA Ab in BAL fluid.

### Splenic GC responses are not necessary for protection against recall challenges

**Figure 6:**
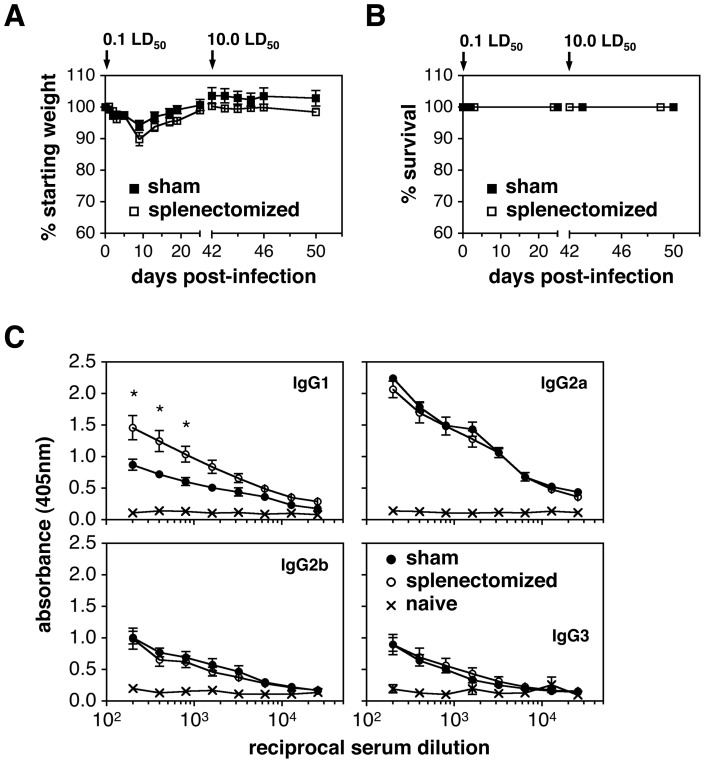
Splenectomized mice withstand a secondary 10.0LD_50_ IAV infection. Sham or splenectomized animals were given a sublethal 0.1LD_50_ dose of IAV i.n. on day 0 and a subsequent 10.0LD_50_ dose of IAV i.n. on day 42. Morbidity and mortality were monitored through day 50. Serum samples were harvested on day 28 following primary sublethal infection. A) Line graphs represent percent of starting body weight (morbidity) after primary and secondary infection. B) Line graphs represent survival (mortality) after primary and secondary infection. C) IgG1, IgG2a, IgG2b and IgG3 Ab responses were measured in day 28 post-infection sera using an IAV-specific whole virus ELISA. Sera from naïve mice were also tested. Each value represents mean ± SEM. *n* = 4 for sham splenectomized mice. *n* = 5 for splenectomized mice. *p<0.05; represents the statistical difference between sham and splenectomized mouse sera at the indicated dilutions as determined by the unpaired Student's t test.

When examining the collective GC response after i.n. challenge with IAV, it is of interest that the largest number of GC B cells was found in the spleen (Figure 1). The phenomenon of B cell [26],[37],[38],[40],[43],[45],[50] and T cell responses in the spleen [5],[9],[10],[60] after respiratory infection with IAV is well known and is likely due to the rapid migration of antigen bearing cells from the lung or dLNs to the spleen [1],[61],[62]. Since GC responses are critical for the generation of long-lived humoral immunity and defense against secondary infection, studies were performed to assess the importance of splenic responses induced during a primary infection in protecting mice against a recall challenge with lethal doses of IAV. In these experiments, splenectomized or sham-splenectomized mice were given a sublethal 0.1LD_50_ dose of PR8 at day 0 followed by a lethal 10.0LD_50_ dose at day 42. Although previous studies have examined the primary response to IAV after splenectomy [43],[63], there are no reports assessing the ability of asplenic mice to withstand a lethal recall challenge. As shown in Figure 6A and 6B, all sham-surgery mice survived the secondary 10.0LD_50_ dose with no weight loss. Similarly, all splenectomized mice withstood the lethal recall challenge without weight loss (Figure 6A and 6B). Since splenectomized mice survived the secondary 10.0LD_50_ dose with little difficulty, IAV-specific Ab levels were determined from sera taken 28 days after primary infection (Figure 6C). The results showed that IgG2a, IgG2b, and IgG3 titers were similar in sera from splenectomized and sham-treated animals. IAV-specific IgG1 levels were modestly higher in splenectomized mice compared to their sham counterparts (Figure 6C). Thus, although the spleen is a site where the largest number of GC B cells is induced after respiratory IAV infection, this organ is apparently not essential for generation of protective Abs during primary infection and thus survival upon lethal secondary challenge.

### IAV-induced T_FH_ cells

**Figure 7:**
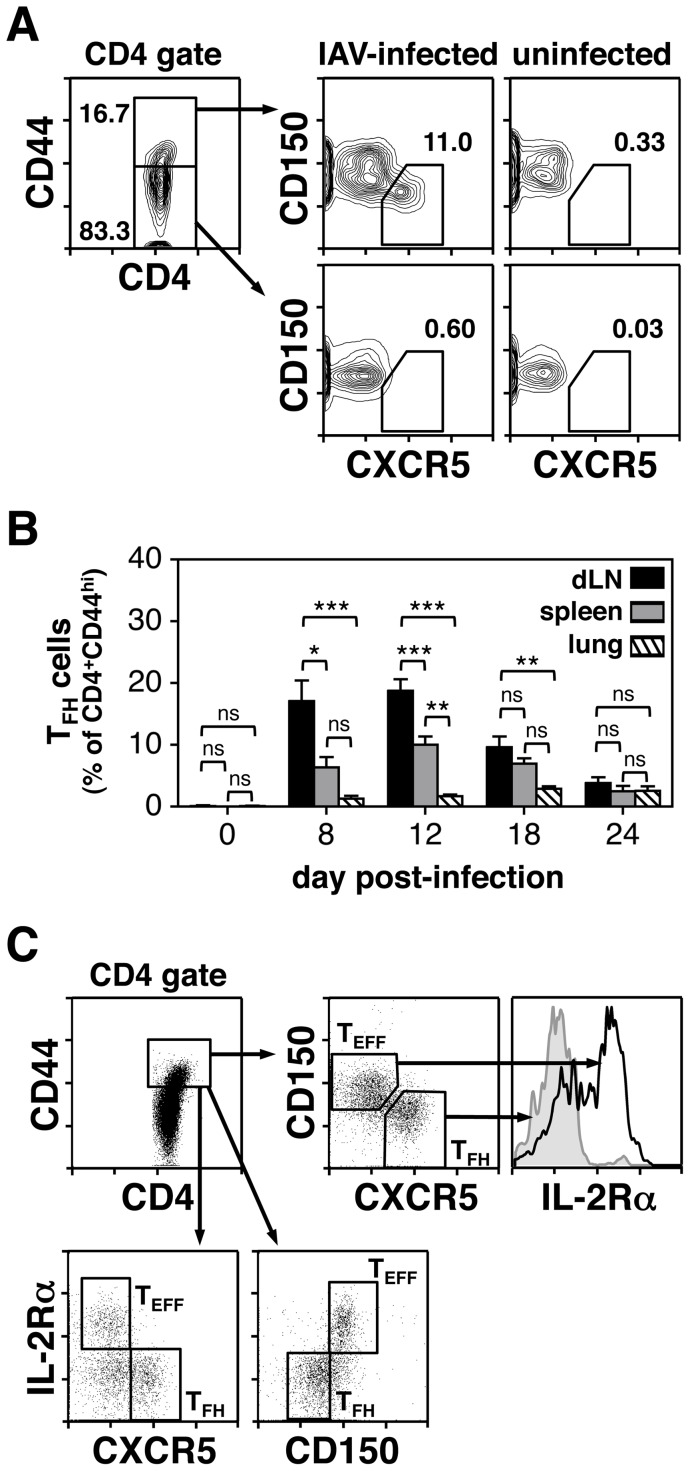
T_FH_ cell populations are induced upon IAV infection. Animals were infected i.n. with a 0.1LD_50_ dose of IAV on day 0. dLNs, lung, and spleen were harvested on days 8–24 post-infection and stained with anti-CD4, anti-CD44, anti-CXCR5 and anti-CD150 mAbs. Tissues from uninfected animals were also analyzed and are shown as day 0. A) Representative plots show the gating strategy used to define CD4^+^CD44^hi^ CXCR5^+^CD150^lo^ T_FH_ cells. The value next to each gate represents the individual percentage from that sample. B) Bar graphs represent the percent of CXCR5^+^CD150^lo^ T_FH_ cells within the CD4^+^CD44^hi^ population from dLNs, spleen, and lung. Each bar represents mean ± SEM. *n* = 5–6 mice for infected mice. *n* = 3 for uninfected mice. ns = not significant; *p<0.05; **p<0.01; ***p<0.001; determined with the ANOVA test. C) Animals were infected i.n. with a 0.1LD_50_ dose of IAV on day 0. dLNs were harvested on day 12 post-infection and stained with anti-CD4, anti-CD44, anti-CXCR5, anti-CD150 and anti-IL-2Rα (CD25) mAbs. Representative plots demonstrate the presence of IL-2Rα^hi^ T_EFF_ cells within the CD4^+^CD44^hi^CXCR5^−^CD150^hi^ population. IL-2Rα^hi^ T cells are nearly absent in the CD4^+^CD44^hi^CXCR5^+^CD150^lo^ T_FH_ gate.

Recent work has demonstrated T_FH_ cells to be critical for the induction and maintenance of GCs [30]–[32]. Given the variability of GC responses in the dLNs, lung and spleen after IAV infection, the next set of experiments evaluated the T_FH_ population in these sites at various time points post-challenge (Figure 7 and Table 1). Based on the work of Crotty and co-workers [64], T_FH_ cells were identified as CD4^+^CD44^hi^CXCR5^+^CD150^lo^ as illustrated in Figure 7A. This figure also shows very low levels of T_FH_ cells in the CD44^lo^ subset of infected mice and an absence in either the CD44^hi^ or CD44^lo^ compartment of uninfected mice. The percentage of CXCR5^+^CD150^lo^ cells within the CD4^+^CD44^hi^ gate was determined for the dLNs, lung and spleen during the course of the response (Figure 7B). The total number of CD4^+^CD44^hi^CXCR5^+^CD150^lo^ cells was also calculated, as was the ratio of total GC B cells to T_FH_ cells (Table 1). When analyzing the percentage of T_FH_ cells within the CD4^+^CD44^hi^ gate, dLNs exhibited the highest level compared to the other organs at days 8 and 12 post-infection (Figure 7B and Table 1). T_FH_ cell proportions were also significantly higher in the dLNs compared to the lung at day 18 post-infection, and significantly increased in the spleen compared to the lung at day 12 (Figure 7B). T_FH_ cells were minimally detected in the lung (Figure 7B and Table 1) and were also very low in NALT (data not shown). The highest total numbers of CD4^+^CD44^hi^CXCR5^+^CD150^lo^ cells were found in the spleen after infection, a result that once again reflects total cell yield (Table 1). It is of interest that the percentages and total cell numbers of T_FH_ cells peaked prior to the height of the GC response in all three sites (Table 1 and Figure 1). This result is consistent with a recent report demonstrating that T_FH_ cells downregulate Bcl6 prior to the peak of the GC response [65]. It is also noteworthy that T_FH_ cells fall to nearly background levels late in the response, when GCs are still clearly present. This latter finding suggests that only a minimal number of T helper cells are required to maintain GC structures after the infection has been resolved. Finally, when examining the ratio of total GC B cells to T_FH_ cells, the lowest value was found in the dLNs, where a nearly 1∶1 ratio was observed at day 8 post-infection (Table 1). This finding may explain why dLNs not only had the highest percent of GC B cells (Figure 1), but also the largest degree of isotype switching throughout the response (Figure 2).

**Table 1:** IAV-induced T_FH_ cell dynamics in dLNs, spleen and lung^1^.

Tissue harvested	Day post- infection	%T_FH_ cells (of CD4^+^CD44^hi^)	Total number of T_FH_ cells^2^ (CD4^+^CD44^hi^CXCR5^+^CD150^lo^)	(Ratio)^3^ GC B cell/T_FH_
dLNs	8	17.1±3.4	6.5×10^3^±2.1×10^3^	1.18±0.4
	12	18.7±1.9	7.0×10^3^±3.0×10^3^	7.1±1.8
	18	9.7±1.7	5.0×10^3^±947	29.8±11.5
	24	4.3±0.6	328±196	221.4±50.8
spleen	8	6.4±1.6	2.4×10^5^±1×10^5^	2.96±0.8
	12	10.1±1.3	2.0×10^5^±7×10^4^	3.55±0.5
	18	6.9±0.9	1.7×10^5^±1.7×10^4^	5.58±1.1
	24	3.3±0.7	9.9×10^4^ ±3×10^4^	7.67±1.7
lung	8	1.3±0.5	4.4×10^3^±1.3×10^3^	9.86±3.4
	12	1.7±0.3	4.6×10^3^±1.1×10^3^	6.02±1.1
	18	2.9±0.4	5.2×10^3^±770	23.4±4.1
	24	2.3±0.4	2.0×10^3^±400	83.8±10.1

1 Animals were given a 0.1LD_50_ dose of IAV i.n. on day 0. dLNs, spleen and lung were harvested on days 8–24 post-infection and T_FH_ cell populations identified by flow cytometry based on their CD4^+^CD44^hi^CXCR5^+^CD150^lo^ phenotype. Values represent mean ± SEM. *n* = 5–6 mice per group and time point.

2 Total number of T_FH_ cells per spleen or lung; total number of T_FH_ cells per node.

3 Ratio of GC B cells to T_FH_ cells is based on total recovered GC and T_FH_ cells per lung or spleen; dLN ratios were based on cell recovery per node.

### Cytokine production from dLN CD4^+^ T cell subsets after IAV infection

In addition to providing co-stimulation signals, T_FH_ cells elaborate cytokines critical for B cell switching and differentiation including IL-21, IL-4 and IFNγ [66]–[70]. A number of recent studies have established IL-21 as a key molecule for optimal T cell-dependent B cell activation and GC formation [71]–[73]. Of interest however, B cell responses to influenza, or immunogens that contain a TLR7 agonist, have been shown to be less dependent on IL-21 [72],[73]. IL-4 and IFNγ are canonical T_H_2 and T_H_1 cytokines, respectively, and important for switching to IgG1 (IL-4) or IgG2 (IFNγ). As demonstrated in Figs. 3 and 4, IgG2a is the dominant isotype expressed on switched GC B cells and IAV-specific antibody secreted in the serum. This is consistent with a T_H_1 bias in response to IAV infection and the production of IFNγ[10],[74]. The question thus arises as to what cytokines T_FH_ cells are producing after IAV challenge, and whether other CD4^+^ T cell subsets contribute to the differentiation of GC B cells.

**Figure 8:**
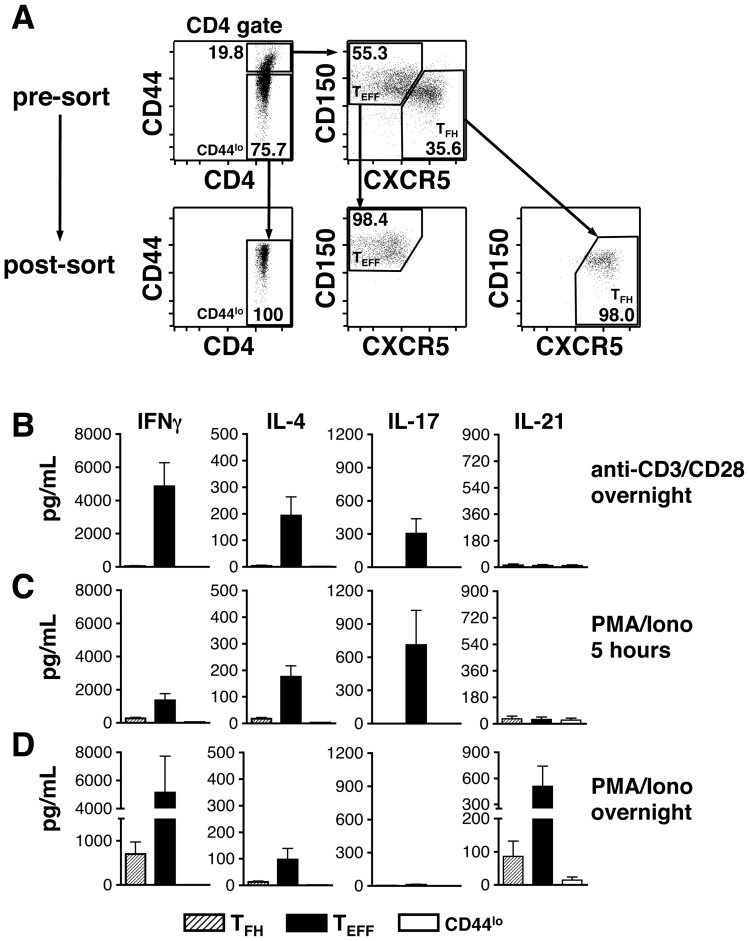
Cytokine production by IAV-induced T_EFF_ and T_FH_ cell populations. Animals were infected i.n. with a 0.1LD_50_ dose of IAV on day 0. dLNs were harvested on day 12 post-infection and stained with anti-CD4, anti-CD44, anti-CXCR5 and anti-CD150 mAbs. The CD4^+^CD44^hi^CXCR5^−^CD150^hi^ (T_EFF_), CD4^+^CD44^hi^ CXCR5^+^CD150^lo^ (T_FH_) and CD4^+^CD44^lo^ (CD44^lo^) populations were sort-purified and stimulated in vitro with anti-CD3 and anti-CD28 mAbs (18 hours) or with PMA and ionomycin (5 hours or 18 hours). Culture supernatants were then harvested and tested for IFNγ, IL-4, IL-17A, and IL-21 protein levels using cytokine bead array kits. A) Representative plots show the pre-sort and post-sort CD44^lo^, T_EFF_ and T_FH_ subsets. B) Cytokine production after overnight culture with plate bound anti-CD3 and anti-CD28 mAbs. C) Cytokine production after 5 hour culture with PMA and ionomycin. D) Cytokine production after overnight culture with PMA and ionomycin. Each bar represents mean ± SEM. *n* = 6 mice. Note that y-axes are identical vertically rather than horizontally in panels B–D.

In addition to T_FH_ cells, it is known that respiratory IAV challenge induces CD4^+^ T effector (T_EFF_) cells with a T_H_1 or T_H_17 phenotype [74]. To confirm the presence of T_EFF_ cells after infection, IL-2Rα (CD25) expression was examined on dLN CD4^+^CD44^hi^ T cells. IL-2Rα has recently been shown to be highly expressed on CD4^+^, CXCR5^lo^, Bcl6^lo^ and Blimp1^hi^ T_EFF_ cells, and low on T_FH_ cells [75]. As shown in Figure 7C, a population of IL-2Rα^hi^ cells was observed within the CD4^+^CD44^hi^ gate. These cells were CXCR5^−^ and CD150^hi^ confirming their T_EFF_ rather than T_FH_ identity. Given the presence of IAV-induced T_FH_ and T_EFF_ cell populations, the next set of experiments tested the key cytokines produced by these CD4^+^ T cell subsets. Lung dLNs were used for these studies given their key role in respiratory immunity and the high percentage of T_FH_ and T_EFF_ cells found in dLNs after i.n. IAV challenge. Initial experiments attempted to document cytoplasmic accumulation of cytokines by flow cytometry in T_FH_ and T_EFF_ cells after culture of whole dLN suspensions. However, this approach proved difficult given the loss of CXCR5 and gain of CD150 expression on CD4^+^ T cells after 5 hours of stimulation. T_FH_ and T_EFF_ populations were therefore sort-purified from freshly isolated dLN suspensions, stimulated in vitro and the culture supernatants were harvested. CD4^+^CD44^lo^ cells were also sorted and served as an antigen-inexperienced internal control population. IL-4, IL-17A, IL-21 and IFNγ protein levels were measured using cytokine bead array kits. The sorting strategy for the three CD4^+^ T cell subsets and a representative post-sort analysis are shown in Figure 8A. Purified T cell populations were stimulated using three different protocols. Specifically, cells were treated overnight with plate bound anti-CD3 and anti-CD28 mAbs (Figure 8B), 5 hours with PMA/ionomycin (Figure 8C) or overnight with PMA/ionomycin (Figure 8D). As expected, CD4^+^CD44^lo^ cells produced virtually no cytokines in all three stimulation protocols. The results further demonstrated that T_EFF_ cells were the most efficient cytokine producers regardless of stimulation regimen. When examining the various cytokines released by the T_EFF_ subset, IFNγ was consistently found in the highest concentration confirming a T_H_1 bias. T_EFF_ cells also produced small amounts of IL-4 after all three activation protocols, and intermediate levels of IL-17A when stimulated overnight with anti-CD3 and anti-CD28 mAbs or with PMA/ionomycin for 5 hours. Curiously, only residual levels of IL-17A were detected after overnight treatment with PMA/ionomycin, suggesting this cytokine to be labile after extended periods in culture. Of interest, significant levels of released IL-21 protein were found only after overnight stimulation with PMA/ionomycin, with the highest levels coming from the T_EFF_ population. T_FH_ cells did generate IL-21 protein, albeit at a modest level, and were in fact better producers of IFNγ. Taken together, these results suggest that both T_FH_ and T_EFF_ cells are capable of contributing to the differentiation of B cells after IAV challenge.

## Discussion

T cell-driven B cell responses result in long-lived high affinity Abs which provide a powerful defense against infectious organisms, including IAV. GC reactions elicit the cellular products that generate these Abs, and form the immunologic basis for inactivated vaccines that protect against seasonal IAV infections. While previous work examining T cell-dependent B cell responses after IAV infection focused primarily on AFCs and Abs, the current study is the first to systematically examine GC responses in the dLNs, lung, spleen and NALT, as well as the T_FH_ cell populations necessary for GC reactions.

After i.n. challenge with IAV, GC reactions were induced in the dLNs, lung, spleen and NALT. Examination of the kinetics, magnitude and isotype distribution revealed variability in the response between the different organs. Whereas GCs were apparent in all sites by day 8 post-challenge, the response peaked at day 12 in the NALT, day 18 in the dLNs and spleen, and day 24 in the lung (Figure 1). These kinetics likely reflect not only antigen availability in the various sites, but the pace at which T_FH_ and B cells are activated, and in the case of the lung, the need to generate organized lymphoid aggregates, or iBALT [42]. On a percentage basis, dLNs exhibited the strongest GC response with nearly 20% of the total recovered cells displaying a GC B cell phenotype at its peak (Figure 1). dLNs also contained a significantly higher percentage of switched (IgM^−^) GC B cells (approximately 60%) compared to those in the lung at every time point and to those in the spleen well into the third week post-infection (Figure 2B and Figure S2). In contrast, IgM^+^ non-switched B cells constituted a significant portion of the GC pool throughout the response (approximately 50%) in the lung and NALT, and during the first 3 weeks in the spleen (Figure 2). Site-specific regulation of the GC reaction after IAV challenge is consistent with previous reports demonstrating organ-specific variability in AFC responses [25],[26],[37]–[41].

When examining the subclass distribution of switched GC B cells after IAV infection, IgG2 (IgG2a + IgG2b) was found to be the dominant isotype in the dLNs, lung and spleen with IgG1^+^ and IgG3^+^ cells constituting smaller subsets (Figure 3B and Figure S3). This observation underscores the T_H_1 nature of the response and is consistent with the distribution of IgG AFCs in these sites after infection [25],[28],[37]–[39]. Of interest, IgA^+^ GC B cells were rare at all time points in the dLNs, lung and spleen (Figure 3 and data not shown). Although this might be expected in the dLNs and spleen, the paucity of IgA^+^ GC B cells in the lung was surprising given the number of IgA^+^ AFCs found in this organ after IAV infection [25],[28],[29],[37],[38]. Further experiments revealed that IgA^+^ GC B cells were easily demonstrable in the NALT (10–15% of the GC population), although IgM^+^ and IgG^+^ cells represented the major GC B cell subsets in this site (Figure 5). The presence of IgA^+^ GC B cells in the NALT suggests that lung IgA AFCs may derive in part from this tissue. It is also possible that IgA AFCs arise in the lung after infection by switching of activated B cells to IgA in a GC-independent manner. T cell-independent switching to IgA has been well characterized in the gut mucosa [76], and induction of IAV-specific IgA Abs in the upper airway has been shown to occur in the absence of cognate B cell-T cell interactions [39].

Although dLNs had the highest GC response as measured by percent of total recovered cells, the spleen contained the largest total number of recovered GC B cells after IAV infection. The kinetics of the splenic GC response was similar to that in the dLNs, indicating rapid transport of viral antigen from the lung to the spleen, most likely by migrating APCs [1],[61],[62]. The marked GC response in the spleen further suggests that this organ may be a key participant in adaptive immune responses after pathogen invasion in the respiratory track, and contribute to long-term humoral immunity and protection against secondary challenge. In order to address this question, splenectomized and sham-splenectomized mice were infected with a sublethal 0.1LD_50_ dose of IAV and challenged 42 days later with a lethal 10.0LD_50_ dose of the same virus. Both groups survived the primary and lethal recall challenge doses, and showed similar morbidity curves (Figure 6). Thus, although the spleen is clearly a participant in the adaptive response to IAV, it is not essential for long-term immunity. These results are consistent with studies demonstrating that upon vaccination, splenectomized humans generated influenza-specific Abs comparable to normal individuals, and were fully protected [77],[78].

In addition to examining the GC response, experiments also tested for the presence of T_FH_ cells in the dLNs, lung, spleen and NALT at multiple time points after infection. T_FH_ cells were detected based on their CD4^+^CD44^hi^CXCR5^+^CD150^lo^ phenotype [64], and similar to GC B cells, the magnitude and kinetics of the T_FH_ cell response differed between organs. When examining the percentage of T_FH_ cells within the CD4^+^CD44^hi^ T cell compartment, dLNs exhibited the highest value at early time points followed by a marked drop as the response matured (Figure 7B and Table 1). This was reflected in the ratio of GC B cells to T_FH_ cells in dLNs with an approximate 1∶1 ratio at day 8 post-infection, and higher ratios thereafter (Table 1). T_FH_ cell values were intermediate in the spleen and very low in the lung and NALT (Figure 7B, Table 1 and data not shown). It is noteworthy that dLNs had significantly higher percentages of GC B cells by day 12 post-infection (Figure 1D) as well as significantly higher percentages of switched (IgM^−^) GC B cells for much of the response compared with the lung and spleen (Figure 2B and Figure 2S). This likely resulted from the degree of T_FH_ cell induction early after infection when GCs were being established, and is consistent with previous work showing that the magnitude of the GC response is directly linked to the size of the induced T_FH_ cell pool [79]. The relatively low percentage of T_FH_ cells in the lung and NALT may reflect the need for these effector cells to migrate from other sites, such as the dLNs and spleen. Regardless of the organ examined, it is clear that numbers of T_FH_ cells were at their maximum prior to the peak of the GC response, and were quite low at late time points when GCs were still present (Figure 1B and Table 1). This finding can be explained by the need for higher numbers of T_FH_ cells during the inductive phase of the GC reaction and minimal numbers for GC maintenance.

T_FH_ cells have been reported to provide a range of signals important for activation and differentiation of GC B cells [30]–[32]. These include co-stimulatory signals delivered through ICOS and CD40L, as well as a number of cytokines [30]–[32]. Although IL-21 is a key molecule produced by T_FH_ cells [66], this subset is capable of producing other cytokines depending upon the challenge antigen [67]–[70]. Experiments were performed to determine which cytokines are produced by dLN T_FH_ cells after IAV infection. Initial attempts utilized a standard approach whereby dLN suspensions were stimulated for 5 hours with PMA/ionomycin followed by staining for T_FH_ cell surface markers and cytoplasmic cytokines. However, short term in vitro activation led to a rapid loss of CXCR5 and gain of CD150, making identification of T_FH_ cells equivocal. IAV-induced dLN T_FH_ cells were therefore sort-purified and stimulated in vitro using a number of activation protocols (Figure 8). T_EFF_ cells from the same dLNs were also sort-purified and stimulated, and cytokine levels in culture supernatants were determined using sensitive bead array kits. This approach thus tested the potential of purified T cell subsets to produce a number of cytokines (IL-4, IL-17, IL-21, IFNγ) upon activation, and also measured production of protein rather than RNA. The results demonstrated that T_FH_ cells produced little to no IL-4 or IL-17, modest levels of IL-21 and higher amounts of IFNγ. The production of IL-21 by this subset is expected, as is the release of IFNγ when T_FH_ cells are induced under T_H_1 conditions [69], [70]. Of interest, T_EFF_ cells were much more potent in their ability to generate cytokines. Not only did this subset produce IL-4 and IL-17, but also released the highest levels of IL-21 and IFNγ when compared with T_FH_ cells. These results suggest the sorted T_EFF_ population to contain a mix of differentiated T helper cell subsets, including T_H_1, T_H_17 and even T_H_2 cells. In addition, the production of IL-21 by T_EFF_ cells is consistent with previous work demonstrating this cytokine to be produced by a range of T cell subsets, including T_H_1, T_H_2 and T_H_17 cells [80], [81]. Finally, the presence of cytokine producing T_EFF_ cells after IAV infection suggests these cells may contribute to the makeup and character of GC responses. Although they are incapable of migrating into follicles, due to the absence of CXCR5, they are likely to influence both T cell-B cell and T cell-DC interactions that occur in the T cell zone or at T cell-follicle borders, and hence activated T cells and B cells that eventually seed the GC reaction.

## Supporting Information

Figure S1 **The majority of B cells in naïve dLNs, lung, spleen and NALT express a follicular phenotype.** dLNs, lung, spleen and NALT were harvested from naïve animals and stained with anti-IgM and anti-CD21 mAb. The contour plots are representative of 3 mice.
(TIF)

Figure S2 **dLNs contain the highest percent of switched GC B cells following IAV infection.** Animals were infected i.n. with a 0.1LD_50_ dose of IAV on day 0. dLNs, lung, and spleen were harvested on days 8–30 post-infection and stained with PNA, anti-B220 mAb and anti-IgM mAb. Bar graphs represent the percent of switched (IgM^−^) B220^+^PNA^hi^ GC B cells from each organ. ANOVA tests were performed comparing IgM^−^ percentages between the different organs at each time point post-infection. Each bar represents mean ± SEM. *n* = 5–6 mice per group and time point. ns =  not significant; *p<0.05; **p<0.01; ***p<0.001.
(TIF)

Figure S3 **The switched GC B cell compartment is dominated by IgG2^+^ cells after IAV infection.** Animals were infected i.n. with a 0.1LD_50_ dose of IAV on day 0. dLNs, lung, and spleen were harvested on days 8–30 post-infection and stained with PNA, anti-B220 mAb and either goat anti-mouse IgG1, IgG2a, IgG2b, or IgG3 specific Abs. Bar graphs represent the percent (left panels) or total recovered cells (right panels) of B220^+^PNA^hi^ GC B cells expressing IgG2, IgG1, or IgG3. “IgG2^+^” refers to the combination of GC B cells expressing either IgG2a or IgG2b. ANOVA statistical tests were performed at each time point comparing IgG2^+^ values with IgG1^+^ and IgG3^+^ (*p* values are annotated as asterisks on the graphs). ANOVA was also applied to determine the extent to which IgG1 increased over time within each organ. IgG1^+^ percentages increased significantly over time in the dLN and spleen, though statistical significance was not achieved when evaluating total IgG1^+^ cell recoveries (IgG1 ANOVA *p* values are not annotated on the graphs). Each bar represents mean ± SEM. *n* = 5–6 mice per group and time point. ns =  not significant; *p<0.05; **p<0.01; ***p<0.001.
(TIF)
