# Supplementary figures and images for: Pulmonary Infection with Influenza A Virus Induces Site-Specific Germinal Center and T Follicular Helper Cell Responses
Source: PLoS One. 2012 Jul 11;7(7):e40733. doi: 10.1371/journal.pone.0040733 (PMC3394713; doi:10.1371/journal.pone.0040733)

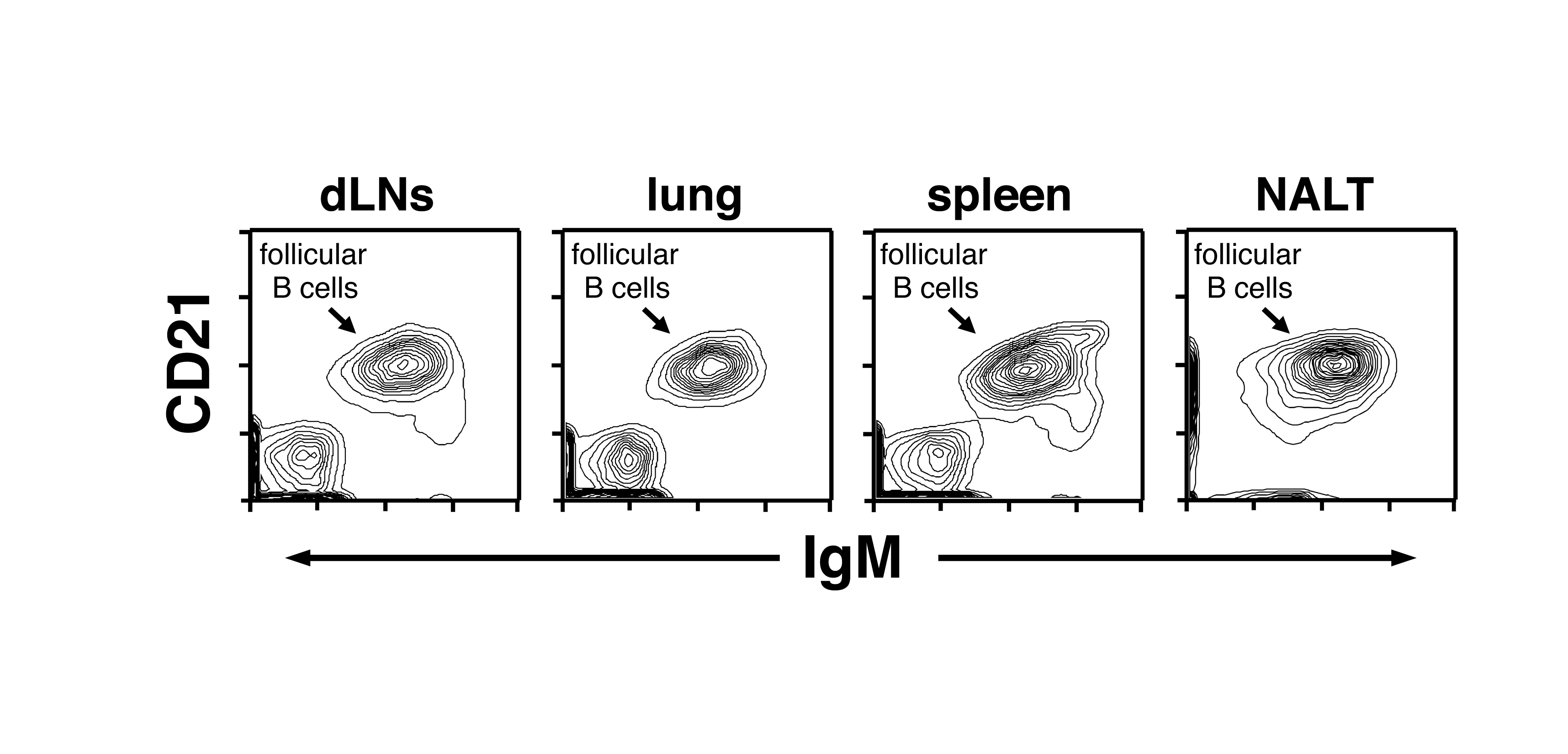

Supplement: Figure S1 — The majority of B cells in naïve dLNs, lung, spleen and NALT express a follicular phenotype. dLNs, lung, spleen and NALT were harvested from naïve animals and stained with anti-IgM and anti-CD21 mAb. The contour plots are representative of 3 mice. (TIF) [file pone.0040733.s001.tif]

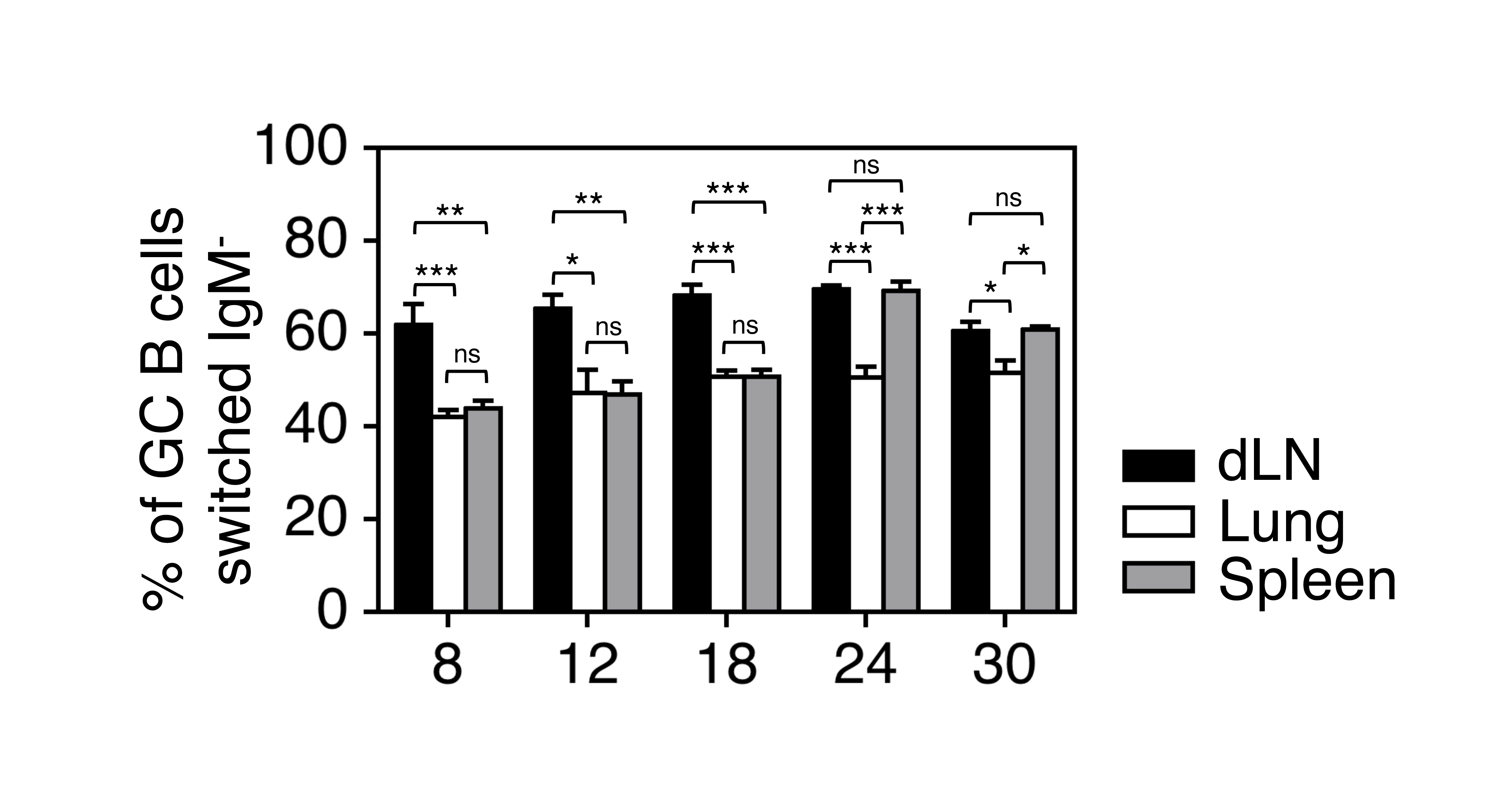

Supplement: Figure S2 — dLNs contain the highest percent of switched GC B cells following IAV infection. Animals were infected i.n. with a 0.1LD50 dose of IAV on day 0. dLNs, lung, and spleen were harvested on days 8–30 post-infection and stained with PNA, anti-B220 mAb and anti-IgM mAb. Bar graphs represent the percent of switched (IgM−) B220+PNAhi GC B cells from each organ. ANOVA tests were performed comparing IgM− percentages between the different organs at each time point post-infection. Each bar represents mean ± SEM. n = 5–6 mice per group and time point. ns = not significant; *p<0.05; **p<0.01; ***p<0.001. (TIF) [file pone.0040733.s002.tif]

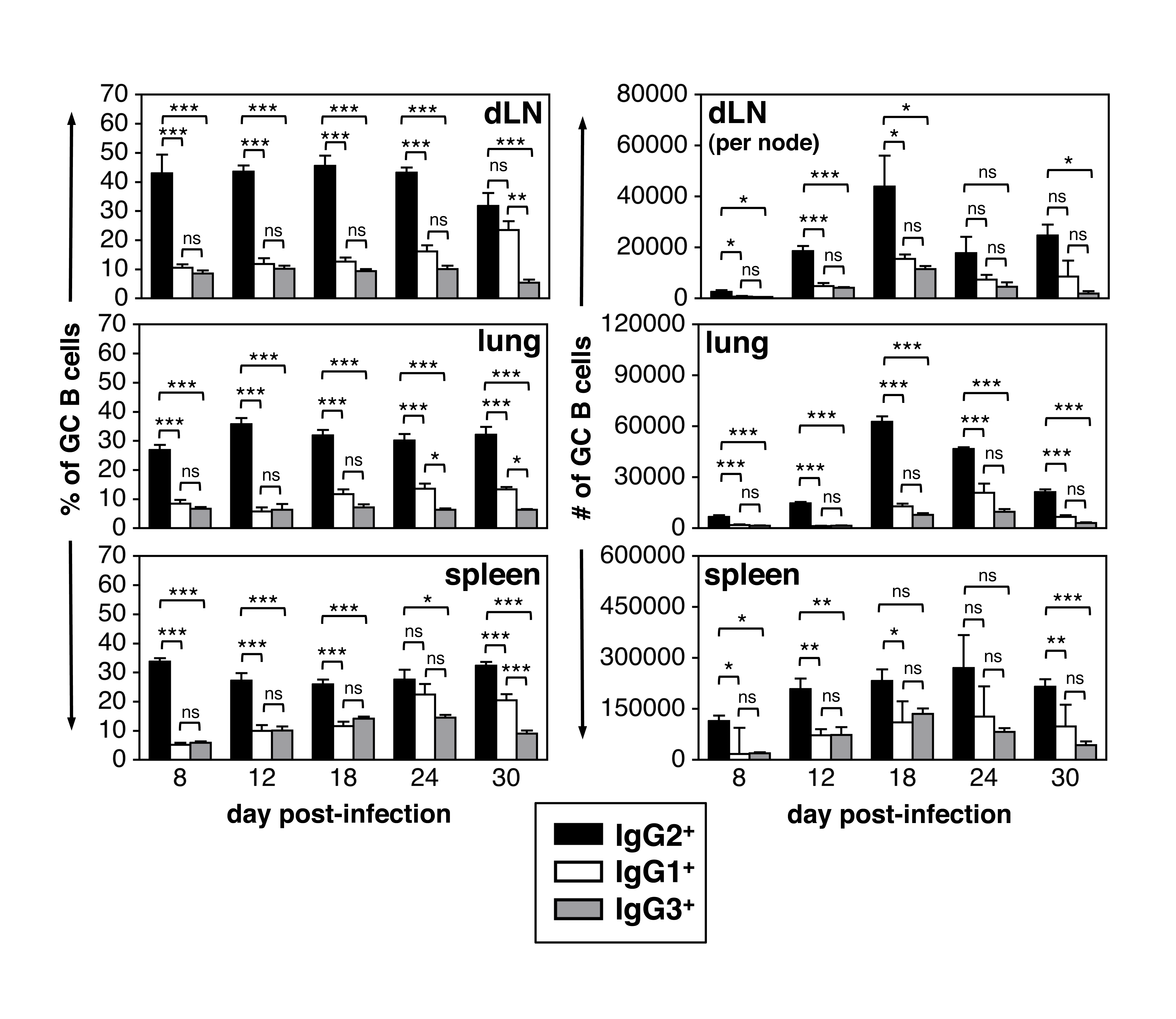

Supplement: Figure S3 — The switched GC B cell compartment is dominated by IgG2+ cells after IAV infection. Animals were infected i.n. with a 0.1LD50 dose of IAV on day 0. dLNs, lung, and spleen were harvested on days 8–30 post-infection and stained with PNA, anti-B220 mAb and either goat anti-mouse IgG1, IgG2a, IgG2b, or IgG3 specific Abs. Bar graphs represent the percent (left panels) or total recovered cells (right panels) of B220+PNAhi GC B cells expressing IgG2, IgG1, or IgG3. “IgG2+” refers to the combination of GC B cells expressing either IgG2a or IgG2b. ANOVA statistical tests were performed at each time point comparing IgG2+ values with IgG1+ and IgG3+ (p values are annotated as asterisks on the graphs). ANOVA was also applied to determine the extent to which IgG1 increased over time within each organ. IgG1+ percentages increased significantly over time in the dLN and spleen, though statistical significance was not achieved when evaluating total IgG1+ cell recoveries (IgG1 ANOVA p values are not annotated on the graphs). Each bar represents mean ± SEM. n = 5–6 mice per group and time point. ns = not significant; *p<0.05; **p<0.01; ***p<0.001. (TIF) [file pone.0040733.s003.tif]
